# Exploring the Potential of Cell-Free Protein Synthesis for Extending the Abilities of Biological Systems

**DOI:** 10.3389/fbioe.2019.00248

**Published:** 2019-10-11

**Authors:** Khushal Khambhati, Gargi Bhattacharjee, Nisarg Gohil, Darren Braddick, Vishwesh Kulkarni, Vijai Singh

**Affiliations:** ^1^Department of Biological Sciences and Biotechnology, Institute of Advanced Research, Gandhinagar, India; ^2^Department of R&D, Cementic S.A.S., Genopole, Paris, France; ^3^School of Engineering, University of Warwick, Coventry, United Kingdom

**Keywords:** CFPS, therapeutics, biocontainment, synthetic biology, virus-like particles, high-throughput proteins

## Abstract

Cell-free protein synthesis (CFPS) system is a simple, rapid, and sensitive tool that is devoid of membrane-bound barriers, yet contains all the mandatory substrates, biomolecules, and machineries required for the synthesis of the desired proteins. It has the potential to overcome loopholes in the current *in vivo* production systems and is a promising tool in both basic and applied scientific research. It facilitates a simplified organization of desired experiments with a variety of reaction conditions, making CFPS a powerful tool in biological research. It has been used for the expansion of genetic code, assembly of viruses, and in metabolic engineering for production of toxic and complex proteins. Subsequently, CFPS systems have emerged as potent technology for high-throughput production of membrane proteins, enzymes, and therapeutics. The present review highlights the recent advances and uses of CFPS systems in biomedical, therapeutic, and biotechnological applications. Additionally, we highlight possible solutions to the potential biosafety issues that may be encountered while using CFPS technology.

## Introduction

Synthetic biology has emerged and continues to grow as a burgeoning scientific field that combines engineering principles with the biological sciences. It is defined as the “design and construction of synthetic biological parts, devices, and systems that do not exist in nature, and also includes the redesigning of natural systems for biotechnological applications” (Endy, 2005; Khalil and Collins, 2010; Qi and Arkin, 2014; Singh, 2014a). In the past decade, a number of synthetic promoters, ribosome binding sites, synthetic genes, scaffolds, and transcription factors have been designed and characterized in a wide range of organisms and cell types (Lutz and Bujard, 1997; Alper et al., 2005; Pfleger et al., 2006; Win and Smolke, 2008; Salis et al., 2009). Similarly, synthetic oscillators (Elowitz and Leibier, 2000; Stricker et al., 2008; Danino et al., 2010), toggle switches (Gardner et al., 2000; Atkinson et al., 2003), biological gates (Tamsir et al., 2011; Moon et al., 2012; Shis and Bennett, 2013; Singh, 2014b), riboregulators (Isaacs et al., 2004; Na et al., 2013), and riboswitches (Tucker and Breaker, 2005; Blount and Breaker, 2006; Patel et al., 2018) have also been designed and characterized in many organisms.

Synthetic systems have been employed in a wide range of biotechnological applications, including the sensing of cancer cells (Culler et al., 2010; Nissim and Bar-Ziv, 2010), toggle switches for controlling metabolic flux (Soma et al., 2014), oscillators for periodic gene expression (Sowa et al., 2014), T-cell controllers (Chen et al., 2010), artificial insemination (Kemmer et al., 2011), and many more. Synthetic chromosomes (Gibson et al., 2009, 2010; Kosuri et al., 2010; Kim et al., 2012; Hutchison et al., 2016) and multiplex automated genome engineering (Wang et al., 2009, 2012; Isaacs et al., 2011; Lajoie et al., 2013; Rovner et al., 2015) have also been developed by utilizing the synthetic biology toolboxes.

The reach of synthetic biology-mediated genome engineering has been significant in a wide range of organisms that currently include bacteria, viruses, yeast, *Drosophila*, zebrafish, and mammalian cells (Cong et al., 2013; DiCarlo et al., 2013; Hwang et al., 2013; Jiang et al., 2013; Mali et al., 2013; Port et al., 2014; Ren et al., 2014; Hisano et al., 2015; Jakočiunas et al., 2015; Zhu et al., 2015; Singh et al., 2017, 2018). With increasing global awareness of health, energy, and environmental issues, prioritizing the exploration and application of synthetic biology has become inevitable, given the potential options that these new sciences could offer. Recent advances in synthetic biology tools have extended their use in basic sciences, biomedical sciences, biotechnology, and industries. This expansion and development become particularly useful for accelerating the invention and innovation in the field of synthetic biology.

The daunting complexity and barrier rendered by the cell membrane prompt numerous difficulties, such as experiment being hard to standardize, incompatibility issues, and variability. To address these issues, cell-free protein synthesis (CFPS) systems, also known as *in vitro* protein synthesis, have emerged as a key tool that can work without the use of living cells. These systems allow one to directly control transcription, translation, and metabolism in an open source fashion (Carlson et al., 2012; Lu, 2017; Moore et al., 2017; Jiang et al., 2018; Yue et al., 2019). CFPS represents a historically important component in the field of biochemistry, duly acknowledging the pioneering effort made by Nobel laureate Eduard Buchner (Nobel Prize in Chemistry 1907) for the discovery of fermentation in yeast cell extracts (YCE) (Buchner, 1897). It has since been repurposed for the understanding of biological processes, most notably contributing to the discovery of genetic code through the use of *Escherichia coli* cell extract by Nirenberg and colleagues (Nirenberg and Matthaei, 1961; Matthaei et al., 1962), which ultimately led them to win and share the Nobel Prize for Physiology or Medicine in year 1968, together with Har Gobind Khorana and Robert Holley.

With the rise of synthetic biology (Gibson et al., 2010), cell-free systems have occupied a scientific niche in helping to develop the understanding of gene networks and biosynthetic pathways (Hodgman and Jewett, 2012; Koch et al., 2018). CFPS requires the core machinery of RNA polymerase, translational apparatus (ribosomes, tRNA synthases, and translation factors), energy-generating molecules, and their cofactors, substrates, and DNA or plasmid templates for obtaining desired products. CFPS has been used for numerous experiments, including the production of proteins that need to be incorporated with toxic amino acids such as canavanine (Worst et al., 2015), incorporation of orthogonal genetic codes (Chemla et al., 2015; Des Soye et al., 2015), production of therapeutics (Zawada et al., 2011), testing of complex gene networks (Shin and Noireaux, 2012; Takahashi et al., 2015a,b), assembly of bacteriophages (Shin et al., 2012), and many more. In the present review, we highlight the recent progress and uses of CFPS in biomedical, therapeutic, industrial, and biotechnological applications.

## Preparation of CFPS Systems

In order to produce a protein of interest, CFPS systems use the components from crude cellular lysates of microorganisms, plants, or animals for sourcing energy and protein synthesis. Commonly used crude extracts are either of *E. coli*, rabbit reticulocytes, wheat germ (WGE), insect cells (Kigawa et al., 2004; Liu et al., 2005; Schwarz et al., 2007), or systems of purified recombinant elements (PURE) (Shimizu et al., 2001; Kuruma and Ueda, 2015), which are commercially available. CFPS system preparation is a simple process, where the cells of interest are grown overnight, diluted, and grown further until the optical density reaches 0.8 to 1.0, after which cells are harvested and sonicated to extract the cell lysate. A buffer mixture augmented with necessary cofactors, energy sources, nucleotides, substrates, amino acids, and tRNAs is added to the cell extract to turn that into a CFPS and cell-free transcription–translation (TX–TL) system (Rustad et al., 2017). A comparison of the advantages of CFPS systems over live-cell counterparts is given in Table 1. Though the price of CFPS systems remains relatively high, it can be reduced by the regeneration of energy and cofactors (Kim and Swartz, 2001; Woodyer et al., 2006).

**Table 1:** Comparison of conventional live cells and CFPS systems.

**Features**	**Live cells**	**CFPS systems**
Genetic manipulation	Complex due to cell membrane barrier	Simple due to open system
Post-transcriptional modification	Simple	Complex
Self-replication	Simple	Complex
Type of DNA template	Plasmids	PCR product or plasmid
Transmembrane protein expression	Complex	Simple
High-throughput biochemical production	Complex	Simple
Incorporation of unnatural amino acids	Complex	Simple
Toxic tolerance	Low	High
Toxic chemical production	Complex	Simple
Gene circuit testing	Complex	Simple
Testing of different chemical concentrations	Complex	Simple
Design–build–test cycles	1–2 weeks	1–2 days
Assembly of virus	Complex	Simple
Testing of orthogonal gene circuits	Complex	Simple
Protein folding	Complex	Simple
Cost	Low	High
Labor	More	Less
Time	More	Less

**Figure 1:**
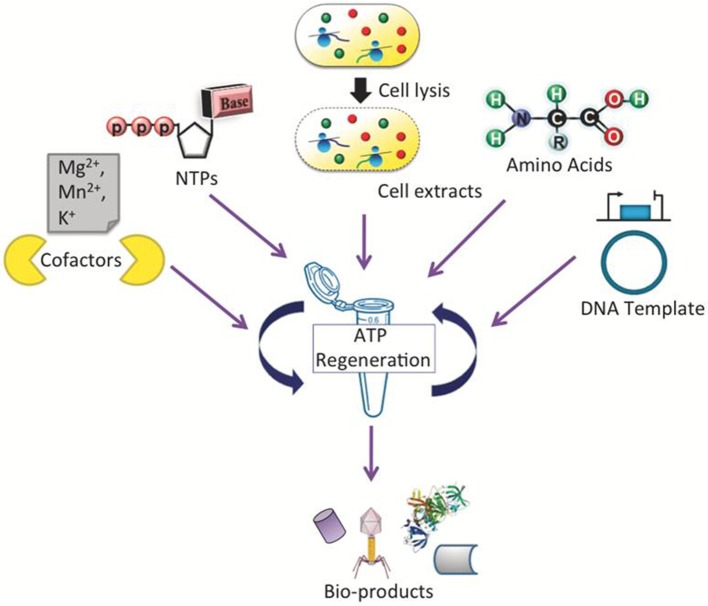
Schematic representation of a CFPS system performed in a single tube which requires cellular lysate, energy sources, nucleotides, amino acids, salts, cofactors, linear or plasmid DNA, and water/buffer to maintain the reaction. Such a system could be used to synthesize viruses, antibodies, therapeutic and high-throughput proteins.

**Figure 2:**
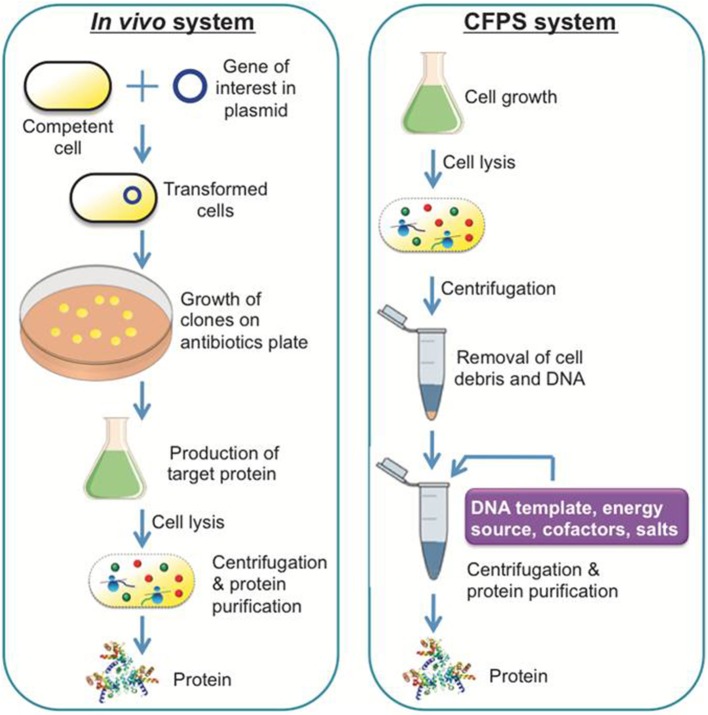
A comparison of a conventional *in vivo* system and a CFPS system. The *in vivo* system is capable of producing recombinant proteins or therapeutic compounds like the CFPS system, although it takes more experimental steps and time to achieve the equivalent result.

A general scheme of a CFPS system is shown in Figure 1, demonstrating a single tube experiment with appropriate buffers containing the requisite cellular lysate and DNA (linear or plasmid), along with associated energy sources, nucleotides, amino acids, salts, and cofactors, that altogether maintain the reaction to generate a product of interest. The products of CFPS can vary across numerous chemical or biological parts, including viruses, therapeutics, antibodies, chemicals, biofuels, and proteins. In producing such chemicals, there are immediate advantages that CFPS systems have over alternative *in vivo* systems, specifically considering the relative speed, simplicity, and effectiveness of the technology. Generally, *in vivo* systems are time consuming and tend to have more steps than the CFPS systems (shown in Figure 2).

Despite this, there is a desire to further reduce the cost and increase product yield of CFPS, especially considering the half-life of reaction, and accordingly researchers have invested their time and efforts to discover alternatives to the compounds that can be used as substrates for protein synthesis in CFPS systems (Zemella et al., 2015). The use of phosphoenolpyruvate (PEP) as an energy source leads to the rapid accumulation of phosphates due to the presence of phosphatase in the cell lysate (Zemella et al., 2015), which in turn leads to a decrease in the amount of ATP from CFPS environment (Calhoun and Swartz, 2005). Accumulation of phosphate is also known to inhibit the protein synthesis in cell-free environments owing to a reduction in the concentration of free magnesium in the reaction system (Kim and Swartz, 1999). Using glucose-6-phosphate in place of PEP as an energy source results in a higher yield of protein in a cell-free environment (Calhoun and Swartz, 2005). Mimicking the physiology of the cytoplasm is another way to increase the protein yield within cell-free systems. Jewett and Swartz (2004) demonstrated that mimicking the pH of cytoplasm and using appropriate buffers in reaction systems increase the yield of protein synthesis when using pyruvate as an energy source. A similar attempt was made to acquire a high yield of proteins while using a cheaper substrate. A study used fructose 1,6-bisphosphate as an energy source for protein synthesis and obtained a titer of 1.3 mg/mL of proteins with an estimated productivity cost at around 0.5 USD per milligram of protein (Kim et al., 2007).

Researchers have designed a protocol that can be easily operated by even non-experts (Levine et al., 2019). The protocol is based upon growing the *E. coli* cells in enriched media using baffled flask and preparing its lysate through sonication. With the addition of appropriate reagents and other substrates, the protocol designed by Levine et al. (2019) was able to give 0.9 mg/mL of super folder green fluorescent protein (sfGFP) in 5 h of reaction time at a cost of 21 USD per milligram of protein synthesized. A study has also been carried out that allows researchers to transcribe and translate the protein of interest in an *in vitro* manner using lysate of thermophilic organism named *Sulfolobus solfataricus* (Lo Gullo et al., 2019). The protocol allows the user to express active protein at high temperature. The optimum temperature to carry out reaction was found to be 70°C without the addition of exogenous components. However, the authors did conclude that the developed protocol is not yet suitable for up-scale production of recombinant protein (Lo Gullo et al., 2019). Due to high rate of protein synthesis found in *Vibrio natriegens*, its extract can act as a potential candidate for high rate of CFPS. Owing to this fact, a highly versatile *V. natriegens* CFPS platform was developed through sonicating the cells, thereby eliminating the need for expensive instruments. A titer of 1.6 ± 0.05 mg/mL of sfGFP was obtained in batch mode CFPS, proving that the *V. natriegens*-based CFPS system was nearly as good as the current CFPS that *E. coli* offers. Upon lyophilization, the active extracts retained the biosynthesis properties and the ability to produce antimicrobial peptides (Des Soye et al., 2018). Similarly, a protocol was also developed that makes use of common lab instruments, and the cell extract can be prepared within 1–2 days using a sonicator. More than 0.26 mg/mL of the sfGFP protein was synthesized within 3 h of reaction setup using the designed protocol by incorporating *V. natriegens* extracts (Wiegand et al., 2019).

Conventionally, studies of membrane proteins are done by obtaining steady yields of target proteins in the form of precipitates, without using membrane-mimicking structures (Zemella et al., 2015). This results in relatively laborious purification and re-solubilization steps that can possibly change the characteristics of the target protein. Membrane-mimicking structures that include detergents (such as Triton X-100 and Digitonin), nanodiscs, and liposomes have been used to facilitate the correct folding of membrane proteins in a cell-free environment (Zemella et al., 2015). The crowding caused by high concentration of macromolecules influences the equilibrium constants and kinetic rates of the experiments including TX–TL-based cell-free experiments (Minton, 2001). Considering this, Rustad et al. (2017) synthesized bacteriophages MS2, ΦX174, and T7 in separate OnePot reactions using an *E. coli*-based cell-free TX–TL system. To mimic better physiology of cytoplasm, they examined the impact by altering the concentrations of magnesium and potassium, as well as by increasing molecular crowding with the addition of PEG 8000 (up to 4.5% wt/vol), which demonstrated dramatic effects on phage synthesis. To further explore the influence of macromolecular crowding in the CFPS system, an equation has been proposed to describe the *in vitro* biomimicry of the crowded environment present within *E. coli* in desire volume of reaction mixture using its lysate (Khambhati et al., 2019a). Sun et al. (2013) have devised a novel method using *E. coli* BL21 Rosetta 2 strain, capable of accessing both the endogenous and exogenous cellular machinery of *E. coli* for protein synthesis. They used bead beating over homogenization and sonication for cell lysis to avoid sample heating and employed the use of 3-phosphoglyceric acid as an energy source, noting higher yield than PEP and creatine phosphate. Compared to the commercially available cell-free systems, this study demonstrated a 98% cost reduction, with material costs of 0.011 USD per microliter of cell reaction, resulting in 0.75 mg/mL of GFP.

With the advancements in CFPS systems, researchers have also attempted to use non-ribosomal biosynthetic pathways for *in vitro* cyclic peptide production. Goering et al. (2016) used PEP as an energy source for synthesizing D-Phe-L-Pro diketopiperazine (DKP), a cyclic dipeptide. DKP was produced by cell-free co-expression of plasmids containing the *GrsA* and *GrsB1* genes. Furthermore, *E. coli* BL21 Star [DE3] strain was used for preparation of cell lysates. In order to convert *GrsA* and *GrsB1* into their functional forms, Bodipy-CoA and Sfp were added directly into the system after incubating the plasmids for 17 h. The system was composed of a single pot experiment that rendered a DKP titer of 0.012 mg/mL. The incorporation of non-canonical amino acids (ncAAs) in polypeptides imparts new functionalities and chemical properties to the target protein, but has remained a difficult task to perform *in vivo* due to its toxicity (Worst et al., 2016). To address this, Worst et al. (2016) created a cell-free TX–TL protocol that allows the successful incorporation of L-canavanine (in place of L-arginine) and L-hydroxy-lysine (replacing L-lysine) into proteins, thus expanding the potential for novel functionalities to be added to proteins while avoiding the risks of cellular toxicity. These studies show that cell-free systems can be a valuable tool for production of complex molecules. A cost-effective OnePot PURE system has been developed, which suggest the researchers to grow 36 essential protein-producing *E. coli* clones in a single flask and purifying them by using single Ni-NTA purification method. The normalized cost improvement of the developed method over the existing PURE system was found to be 14-fold at a cost of 0.09 USD per microliter of reaction with a titer of 0.156 mg/mL of protein (Lavickova and Maerkl, 2019).

## Potential Applications of CFPS Systems

**Figure 3:**
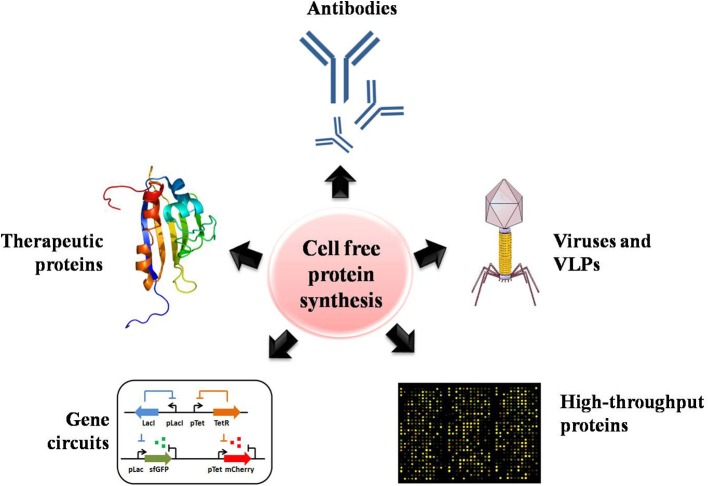
The numerous potential applications of CFPS systems for assisting high-throughput protein production, including the production of antibodies, proteins with unnatural amino acids, therapeutics, viruses and virus-like particles, and gene circuits.

The productivity, cost, scale, and complexity of recombinant proteins have rapidly extended the uses and commercialization of CFPS systems (Swartz, 2006). In this section, we highlight the successes obtained with CFPS systems across numerous projects that have looked at increasing and improving the production of valuable products, including proteins, enzymes and therapeutics. The potential applications of CFPS systems are summarized in Figure 3.

### High-Throughput Proteins

In the post-genomic era, high-throughput CFPS platforms have received much attention due to their numerous advantages which include (i) direct use of PCR templates to avoid exhaustive cloning steps, (ii) cost-effective reactions, (iii) potential for miniaturization as well as automation via microfluidics chips, and (iv) lack of a cell wall barrier that allows easy manipulation of reactions. The use of CFPS systems could be a better choice for labeling, through incorporation of unnatural amino acids (UAAs), which is quite useful for nuclear magnetic resonance (NMR) or X-ray crystallographic analysis (Sawasaki et al., 2002; Jin and Hong, 2018). Accordingly, CFPS has demonstrated efficiency for easy incorporation of labeled amino acids and high protein expression that allows better NMR analysis (Sawasaki et al., 2002; Morita et al., 2003; Ozawa et al., 2004; Takai et al., 2008).

CFPS systems have been used for the large-scale synthesis of protein libraries for functional genomics studies. Protein *in situ* arrays (PISA) have been used for quick and efficient generation of CFPS systems to study protein interaction on biochips (He and Taussig, 2007; He et al., 2008). WGE-based CFPS systems have been used for synthesizing 13,364 human proteins, creating the infrastructure for a human protein factory. Notable results from this include the finding of 58 of the 75 synthesized human phosphatase enzymes to be functional and later printing them onto glass slides to build a functional protein microarray (Goshima et al., 2008).

Chinese hamster ovary (CHO) cell line is recognized as safe and most prominent for industrial protein production. The cell lysate of CHO contains microsomes and may also contain proteins including disulfide isomerase or binding immunoglobin protein that are essential for disulfide bridging and correct folding of disulfide bridged proteins. These features can be used to synthesize proteins that are difficult to express by ensuring continuous-flow cell-free systems of the CHO cell lysate. Optimizing the CFPS reaction conditions produced up to 0.98 mg/mL of membrane protein (Thoring et al., 2017). Apart from CHO cell lysate, cultured *Spodoptera frugiperda* 21 cells have also been used to take advantage of translocationally active microsomes. With the combination of internal ribosome entry sites (IRES) and continuous exchange CFPS reaction protein translation, the epidermal growth factor receptor production reached up to 0.285 mg/mL (Quast et al., 2016). Similarly, 0.7 mg/mL of the virus envelope protein (gp67) was also attained from insect cell lysates (Merk et al., 2015). CFPS systems can be further expanded for expression and testing of higher libraries in 384 well formats, allowing complex studies and high-throughput experiments to work sufficiently faster, quicker, and at a lower cost.

Protein products play a vital role in the field of medical care today. A significant proportion of proteins that are being used in biopharmaceutical and industrial fields are difficult to express, as they are often too complex and toxic, or belong to membrane proteins that are difficult to produce using living cells. Therefore, a primary goal of CFPS system is to regulate and optimize the protein production *in vitro*. High levels of protein toxicity can result in death of living cells during gene cloning and expression *in vivo*. Toxic proteins can interfere with metabolic biosynthetic pathways and tend to inhibit cell division. Therefore, they are hard to express in high amounts *in vivo*. A few of the highly toxic proteins have already been expressed and purified from cell-free systems, including restriction endonucleases (Goodsell, 2002), cytolethal distending toxin (Ceelen et al., 2006), and human microtubule-binding protein (Betton, 2003). Since there is no reliance on cellular growth and division, CFPS systems can be used as an alternative and excellent platform for toxin production. Membrane proteins continue to gain scientific attention due to their potential as drug targets, although *in vivo* overexpression of such proteins remains a critical bottleneck in research progress due to their complex structures, potential toxicity, tedious preparation, and low efficiency. A number of studies have suggested that CFPS systems can be used for overexpression of membrane proteins, with successes demonstrated for a number of membrane proteins that include the G-protein coupled receptor (Orbán et al., 2015), vaccine antigens (Welsh et al., 2012; Lu et al., 2014), and tetracycline pump TetA (Wuu and Swartz, 2008). In this regard, the non-natural and highly toxic (in the context of living cells) amino acid canavanine, an analoge of arginine that could serve as a possible antimetabolite and organic allelochemical agent, has been expressed via the CFPS system (Worst et al., 2015).

**Figure 4:**
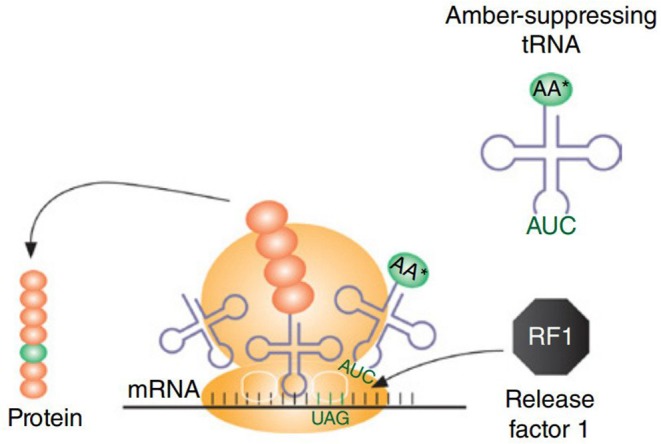
For incorporation of non-natural amino acids, amber-suppressing tRNAs are used. A strain having RF1 would create a competition between amber suppression tRNA and RF1 to bind with site-specific amber codon, which is represented in the figure. To avoid this competitive binding, cell extract is prepared using a strain lacking RF1. Figure reproduced with permission from Schoborg and Jewett (2018) © John Wiley and Sons, Inc.

CFPS system has even been used for incorporation of UAAs using orthogonal tRNA, producing 0.9–1.7 mg/mL of soluble sfGFP variants, containing either p-azido-L-phenylalanine (pAzF) or p-propargyloxy-L-phenylalanine (pPaF), which accumulated in the CFPS solution (Albayrak and Swartz, 2013). Correspondingly, CFPS has been used for incorporation of non-standard amino acids (nsAAs) for the generation of proteins and enzymes with novel properties, renewed structural elements, and prominent functions (Hong et al., 2014). For the cell lysate preparations, *E. coli* cells lacking RF1 (release factor 1), a protein known to terminate the machinery supporting translation, was used. To incorporate site-specific nsAAs, an amber suppression mechanism was used where 13 occurrences of the amber stop codon (UAG) were reassigned with synonymous ochre (UAA) codon (rEc.E13.ΔprfA) (Figure 4). The maximum production was noted to be 0.19 ± 0.02 mg/mL of soluble sfGFP that contained either a single pPaF or p-acetyl-L-phenylalanine (pAcF) in its sequence (Hong et al., 2014). In another study, the site-specific integration of UAAs was used to expand the protein diversity and proteomic code (Shrestha et al., 2014). The cost consumption was reduced by 55% using alternative energy sources (such as glucose). Linear expression templates (LETs) were used for the expression and incorporation of UAAs, as using LET-based systems reduce the labor expense in comparison to *in vivo* or plasmid-based CFPS production. Labor expenses are reduced in terms of the steps required for production. LETs-based system requires only four steps, i.e., PCR, CFPS, purification, and analysis, whereas *in vivo* and plasmid-based CFPS require additional steps that include synthesis of a plasmid library, transformation into an expression strain (for *in vivo* CFPS), plasmid purification (for plasmid-based CFPS), and cell growth along with its maintenance (*in vivo*). In the same perspective, UAAs were incorporated at site-specific locations using the CFPS system of *E. coli* in combination with an aminoacyl-tRNA synthetase and a suppressor tRNA evolved from *Methanocaldococcus jannaschii* which rendered a high titer (up to 1 mg/mL) of proteins bearing the incorporated UAAs at specific sites (Ozawa and Loh, 2014). Subsequently, the crude cell extracts of a genomically recoded *E. coli* strain (MCJ.559) lacking RF1 and disabled for five negative effector nuclease genes (*rna, rnb, csdA, mazF*, and *endA*) were used to produce 0.55 ± 0.04 mg/mL of sfGFP containing pAcF, which was further maximized to 1.3 mg/mL by using a semi-continuous system (Hong et al., 2015).

Engineering tRNAs and aminoacyl-tRNA synthetase for expansion of genetic code through the incorporation of nsAAs is another approach (Martin et al., 2018). A CFPS platform using MAGE in *E. coli* through the deletion of RF1 has been developed (Martin et al., 2018). This optimized platform initially obtained a titer up to 1.78 mg/mL of sfGFP, which upon extension through incorporation of 40 identical pAcF residues into an elastin-like polypeptide produced 0.096 mg/mL of the desired polypeptide having 98% accuracy (Martin et al., 2018). This system could be useful for the incorporation of other UAAs for understanding the novel aspects of protein diversity and functionality. Furthermore, an effective and efficient protocol has been designed for tightly regulated addition of UAA at a particular site of protein (Gao et al., 2019). The components of orthogonal translation system were added separately and orthogonal-tRNAs were added indirectly (Gao et al., 2019).

**Figure 5:**
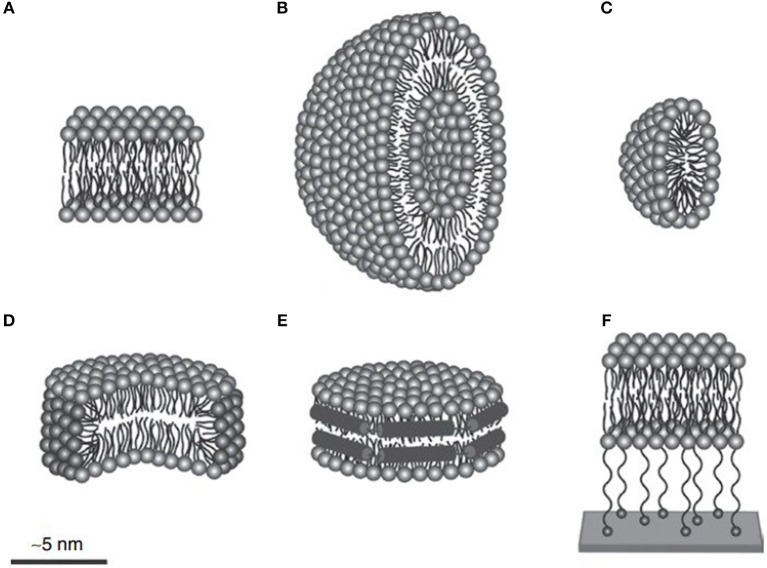
To produce membrane proteins through CFPS platforms, several membrane-mimicking structures are used. **(A)** Lipid bilayer, **(B)** liposome, **(C)** micelle, **(D)** bicelle, **(E)** nanodisc, and **(F)** tethered bilayer lipid membrane. Figure reproduced with permission from (Schoborg and Jewett, 2018) © John Wiley and Sons, Inc.

The yield of membrane protein through cell-free expression is much less compared to non-membrane proteins (Krishnan et al., 2019). Thus, efforts have been made to increase the yield of membrane proteins through cell-free expression by designing a protocol using detergent and lipid nanodisc (Figure 5). Results have revealed that detergents such as Brij-35 and Brij-78 are suitable for solubilizing the photosystem II subunit S (PSBS) membrane protein (Krishnan et al., 2019). One of the reasons for this might be the long chain of polyoxyethylene-alkyl-ethers present in Brij-35 and Brij-78 detergents along with their hydrophilic head that aids the solubilization of the PSBS protein. Moreover, the designed cell-free protocol facilitates the expression of PSBS protein using continuous exchange system at 30°C temperature with a titer of 500 ng/μL of the reaction system. The purification and refolding of the PSBS protein were facilitated by using n-dodecyl β-D-maltoside detergent (Krishnan et al., 2019).

### Therapeutics

Numerous studies have stressed on the substantial functional efficiency of recombinant proteins derived from CFPS systems. CFPS has been accredited to be a viable option for the production of therapeutic proteins (Tran et al., 2018). Complex proteins such as urokinase protease and a variant of the human tissue-type plasminogen activator, containing six and nine disulfide bonds, respectively, have been produced with the help of cell-free systems using *E. coli* cell extracts (Kim and Swartz, 2004; Yin and Swartz, 2004). The therapeutic importance of urokinase protease relates to its activity as a thrombolytic agent that aids the treatment of thrombus-related diseases. The key role of urokinase protease is to convert plasminogen to plasmin, a step that helps to dissolve blood clots (Craik et al., 2011). Similarly, variants of tissue plasminogen activator have the potential to be used as drugs for the treatment of acute ischemic stroke (Zivin, 2009). However, the reducing activity of disulfide in cell lysate makes it difficult to form disulfide bonds in proteins. This problem can be solved by designing an adapted method in which cell extracts when treated with iodoacetamide before initiating protein synthesis reactions, abolish the reducing activity of disulfide present in the cell lysate (Kim and Swartz, 2004; Yin and Swartz, 2004). Furthermore, the use of the purified disulfide isomerase DsbC and a glutathione redox buffer in the reaction system allows the formation of active urokinase protease (0.04 mg/mL) that contains six disulfide bonds in its final expressed structure. Besides this, the human tissue-type plasminogen activator protein with increased solubility and titer (up to 0.06 mg/ml) was attained by using Skp (an *E. coli* periplasmic chaperone) and organic chemicals spermidine and putrescine in the reaction system, into which few other organic chemicals were added to maintain more natural environment (Yin and Swartz, 2004). A summary of the therapeutic proteins that have been produced by CFPS systems is given in Table 2.

**Table 2:** List of therapeutic proteins produced through CFPS.

**Product**	**Cell extract**	**Titer (mg/mL)**	**Potential application**	**Reference**
Single-chain antibody variable fragment against *Salmonella* O-antigen	Wheat Germ Extract	0.013	*In vivo* diagnostic and immunotherapeutic	Kawasaki et al., 2003
Urokinase protease	S30 extract (*E. coli* K12)	0.04	Treatment of thrombus	Kim and Swartz, 2004
Variant of human tissue-type plasminogen activator	*E. coli*	0.06	Treatment of acute ischemic stroke	Yin and Swartz, 2004
38C13B lymphocyte Id scFv	*E. coli* (Cytomim system)	0.043^**^	Lymphoma immunotherapy	Yang et al., 2005
Insulin-like growth factor I	*E. coli*	0.4	Central nervous system disorders (e.g., PMS, Rett syndrome)	Swartz, 2006
Murine granulocyte macrophage-colony stimulating factor (mGM-CSF)	*E. coli* (KC6)	0.854 ± 0.054^*^	Stimulator of systemic anti-tumor immunity	Goerke and Swartz, 2008
hGM-CSF		0.823 ± 0.060^*^	Cancer immunotherapy, healing chronic wounds	
Human granulocyte colony-stimulating factor (hG-CSF)		0.619 ± 0.068^*^	Cancer therapy	
Human Interferon alpha 2b (hIFNα2b)		0.692 ± 0.046^*^	Anti-cancer agent	
Murine scFv (Mvlvh)		0.519 ± 0.038^*^	Vaccines	
Human scFv (Hvlvh)		0.455 ± 0.007^*^		
Fusion protein with [bacterial immunity protein (im9)] Im9-hvlvh		0.441 ± 0.021^*^		
mGM-Im9-mvlvh		0.628 ± 0.056^*^		
mGM-Im9-hvlvh		0.591 ± 0.048^*^		
Human consensus interferon-alpha	*E. coli* (S30)	0.4	Anti-viraland anti-tumor agents	El-Baky et al., 2011
Human granulocyte-macrophage colony-stimulating factor (hGM-CSF)	*E. coli* (KGK10)	0.7	Cancer immunotherapy, healing chronic wounds	Zawada et al., 2011
Onconase	*E. coli* (PANOxSP system)	0.03 (>80% soluble)	Treatments of malignant mesothelioma	Salehi et al., 2016
Botulinum toxins	*E. coli* (RTS-100, RTS-500, and RTS-9000 HY kits)	1	Botulinum vaccine	Zichel et al., 2010
Streptokinase	HeLa and CHO cell lysates	0.50	Thrombolytic therapy	Tran et al., 2018
Crisantaspase	*E. coli* ClearColi	1	Cancer therapy	Wilding et al., 2019

* *Total titer*,

** *Soluble titer*.

The granulocyte macrophage colony-stimulating factor (GM-CSF) fusion proteins are potent B-cell lymphoma immunotherapeutic vaccines (Yang et al., 2005). For displaying the correct biological activity of these proteins, both GM-CSF and the B-cell lymphoma idiotype scFv should form two different disulfide bonds, and the conjugation should be at the amino terminus of GM-CSF to obtain a biologically functional product at the end (Yang et al., 2005). In a constructed CFPS system for production of active conjugates, an *E. coli* cell extract pretreated with iodoacetamide (IAM) containing sulfhydryl redox buffer was used and a titer of 0.043 mg/mL of 38C13 B-lymphocyte Id scFv conjugates was achieved (Yang et al., 2005).

The therapeutic importance of insulin-like growth factor I (IGF-I) for the treatment of some central nervous system disorders, including premenstrual syndrome (PMS) and Rett syndrome in children is immense (Costales and Kolevzon, 2016). *E. coli* cell lysates were used in a CFPS system to produce up to 0.4 mg/mL of IGF-I (Swartz, 2006). The human granulocyte-macrophage colony-stimulating factor (rhGM-CSF) can also act as a therapeutic protein. It has been produced by Sutro Biopharma Inc. using a CFPS system hosted in a volume of 100 liters, which is one of the largest CFPS system used to date. This marks a notable instance that demonstrated the inherent potential of CFPS for the production of therapeutic proteins, and in their CFPS system, they could produce up to 0.7 mg/mL of rhGM-CSF within 10 h of incubation (Zawada et al., 2011). The possible uses of rhGM-CSF include assisting cancer immunotherapy and healing of chronic wounds (Vanitha et al., 2017; Brem et al., 2018).

CFPS is an important platform that can synthesize medically important molecules rapidly in a cost-effective manner; however, the type of CFPS may affect the end result. In this context, two *E. coli* CFPS platforms were established, where one was cell extract-based and the other was a generic cell-free platform. The generic platform showed higher expression of therapeutic proteins, antibody fragments, and vaccines, obtaining titers of 0.71, 0.23, and 0.3 mg/mL, respectively (Goerke and Swartz, 2008). Later, anti-cancer agents hGM-CSF, hG-CSF, and human Interferon alpha 2b (hIFNα2b) were produced up to 0.823 ± 0.060, 0.619 ± 0.068, and 0.692 ± 0.046 mg/mL, respectively (Goerke and Swartz, 2008). The capability to produce a number of potential vaccines was also analyzed, with CFPS-based production of murine scFv (Mvlvh) and human scFv (Hvlvh), along with fusion proteins of bacterial immunity protein (im9) Im9-hvlvh, mGM-Im9-mvlvh, and mGM-Im9-hvlvh, where a titer of 0.519 ± 0.038, 0.455 ± 0.007, 0.441 ± 0.021, 0.628 ± 0.056, and 0.591 ± 0.048 mg/mL, respectively was recorded (Goerke and Swartz, 2008). Apart from that, up to 0.4 mg/mL of the consensus interferon cIFN-α was also achieved with a CFPS system of *E. coli* cell lysate (El-Baky et al., 2011). The potential applications of this compound stems from its capability as an anti-cancer drug, as it exhibited the anticancer effects during their *in vitro* studies (El-Baky et al., 2011).

Earlier, for the production of onconase, a drug for treatment of malignant mesothelioma, live *E. coli* cells were normally subjected to lysis and thereafter the protein was purified from inclusion bodies found in the cell pellets (Salehi et al., 2016). The issue has been addressed by a novel method that comes with an additional advantage of direct and immediate characterization of onconase without the need for laborious purification steps. The method can be used to produce up to 0.03 mg/mL of onconase in an active form with about 80% of the mass being soluble onconase (Salehi et al., 2016).

Streptokinase, an important enzyme in thrombolytic therapy, has been produced up to 0.5 mg/mL, within 2.5 h of incubation through CFPS using HeLa and CHO cell lysates in such a way that the resultant protein was neither glycosylated nor had any disulfide bonds. Following an initial characterization, it was proven to be functionally efficient in terms of activity and outcome. Additionally, the use of an inert purification technology for the purification of proteins returned a better yield compared to standard affinity chromatographic technologies (Tran et al., 2018). 0.013 mg/mL of single-chain antibody variable fragment (scFv) against *Salmonella* O-antigen was produced with the help of CFPS with WGE (Kawasaki et al., 2003). The obtained product may have diagnostic and immunotherapeutic applications owing to its shorter tissue clearance time and reduced immunogenicity (Anand et al., 1991).

WGE has been used for the overexpression of 124 genes from the *Plasmodium falciparum* genome for aiding the development of a malaria vaccine (Tsuboi et al., 2008, 2010). Out of the 124 genes, 93 genes (74%) were expressed in the soluble form. Interestingly, it was found that native codon usage in genes resulted in a higher output when compared to using codon-optimized genes. Similarly, CFPS systems have been used for expressing the botulinum toxins, obtaining a titer up to 1 mg/mL (Zichel et al., 2010). The use of CFPS systems for production of a botulinum vaccine could eliminate the problem of codon bias, faced during the *in vivo* production of the recombinant botulinum toxin Hc chain upon using *E. coli* and the yeast *Pichia pastoris* as host organisms. In addition, a novel CFPS system based on *Saccharomyces cerevisiae* for protein and therapeutic production has been developed. In testing, the system managed to obtain a titer up to 0.007 mg/mL of firefly luciferase in batch reactions. In this system, factors such as expensive reagents and extraneous processing steps were eliminated (Hodgman and Jewett, 2013).

Any therapeutic protein produced from *E. coli* demands extensive and costly purification steps, so that the manufacturer can avoid the accumulation of *E. coli* endotoxin in the final product. The presence of endotoxin in the product can potentially lead to septic shock in the patient (Wilding et al., 2019). CFPS system generated from ClearColi® cells lysate can act as the solution for production of cell-free mediated endotoxin-free therapeutic proteins. ClearColi® cells are devoid of the endotoxin that is usually found in other *E. coli* cells; however, the protocol for extract preparation is slightly different as compared to other *E. coli* strain due to its reduced growth rate and osmolarity sensitivity (Wilding et al., 2019). A study demonstrated the production of crisantaspase from ClearColi® cells lysate containing reduced *E. coli* endotoxin, thus removing the costly and extensive steps for its endotoxin purification. The titer of crisantaspase obtained was comparable to that produced by the extract of *E. coli* BL21 strain, i.e., nearly 1 mg/mL. Crisantaspase is a therapeutic protein that has been approved by FDA for cancer therapy (Wilding et al., 2019).

Glycoproteins have immense therapeutic potential, though it demands extensive as well as costly purification steps, thus posing a challenge for its production (Daniel et al., 2019). These proteins are produced in a cellular compartment called Golgi apparatus where a protein undergoes series of reactions for its glycosylation. Few of the reasons for low yield of protein synthesis through cell-free system are competing reactions and formation of undesired side products (Daniel et al., 2019). However, a cell does not face the mentioned issues as it localizes a particular reaction at a particular place through compartmentalization. Learning from the cells, a Golgi-on-chip technique was devised to achieve compartmentalization in cell-free system for specific synthesis of glycoproteins (Daniel et al., 2019).

### Virus and Virus-Like Particles

Viruses are small infectious agents that are incapable of self-reproduction and use the host machinery for their propagation. Viruses can physically and metabolically remodel the host cell to establish an optimal environment for their replication (Chukkapalli et al., 2012). Viruses being able to infect a wide variety of different cell types, genetically modified viruses transporting foreign DNA have contributed greatly to the development of experimental gene therapy treatments. In comparison to *in vivo* studies, DNA-based CFPS systems provide a tool for the investigation of complex biological systems with a greater degree of control and freedom than other methods (Shin et al., 2012). In order to support the study of complex biological systems, Shin et al. (2012) demonstrated the replication, synthesis, and self-assembly of T7 and ΦX174 bacteriophages *in vitro* to establish the fact that large DNA programs can be efficiently expressed outside the cell (in their case, a test tube) through cell-free TX–TL systems. They managed to assemble around 0.1 to 1 billion functional phages with the use of 1 nM of genome (the target DNA). The first lot of phages began to be synthesized following 1 h of incubation and their accumulation continued for 5 h. It was also reported that the addition of dNTPs increased the phage production by nearly 200-fold and further investigations revealed that after the fourth hour of incubation, genomic DNA began to degrade, contributing to the observed arrest in synthesis. Even though a cytosolic lack of thioredoxin tends to impair DNA replication *in vivo*, it was highlighted that the absence of thioredoxin in these cell-free TX–TL systems does not impede phage production (Shin et al., 2012).

VLPs are multi-protein structures, averaging between 25–100 nm in size, capable of self-assembly and can systematically mimic the conformation of proteins found in the native virus (Roldão et al., 2010; Carlson et al., 2012). These bio-nanomaterials are devoid of genetic information and as such cannot self-reproduce, but due to their ability to display high-density viral surface proteins, they may successfully penetrate into a living cell. These particles are formed during the heterologous expression of viral proteins of the same or different viruses in a system, or spontaneously during the viral life cycle inside a cell (Chroboczek et al., 2014). These empty shells (lacking a viral genome) have the potential to be used as safe vaccines because of their ability to elicit an immune response and lack of self-replication. Stimulation of innate immunity by VLPs is facilitated through pattern recognizing receptors and toll-like receptors and the induction of a strong humoral response. This is augmented further through better uptake, processing, and presentation by antigen-presenting cells, due to highly specific structures and multimeric antigens of VLPs (Shirbaghaee and Bolhassani, 2016). Aside from vaccination, VLPs have gained interest in fields of gene therapy, drug delivery, nanotechnology, and diagnostics (Shirbaghaee and Bolhassani, 2016).

In conventional cell-based VLPs production, they were produced *in vivo* and their assembly was separated from the large pool of proteins *ex vivo*. Numerous difficulties are faced in conventional cell-based system such as poor yields, low solubility of the bacteriophage proteins, lack of post-transcriptional modifications, complications in expressing mammalian viral proteins, less stability of VLPs, costly product formation, and difficulties in the separation of morphologically similar contaminant proteins in different host systems (Pattenden et al., 2005). Accordingly, a number of studies have attempted to address these problems. In a study, Bundy et al. (2008) built an *E. coli-*based CFPS system for VLPs formation. Several advantages were listed over the currently used cell-based systems, including the redirection of metabolic resources more toward *in vitro* transcription and translation, one-step purification as well as recovery, and the removal of the laborious procedures of cell transformation. For enhancing the stability of VLPs, Bundy and Swartz (2011) controlled the redox potential of the reaction system, allowing them to be able to control the formation of disulfide bonds between capsid monomers, thus altering VLP stability. In another study, Patel and Swartz (2011) produced VLPs of bacteriophages Qβ and MS2, which possessed the ability to be linked with azide and alkyne-containing multiple proteins that included the antibody fragment scFv and granulocyte macrophage colony-stimulating factor (GM-CSF) together, nucleic acids and poly-ethylene glycol (PEG) chains. In their experiment, they produced azide and alkyne methionine analogs on the surface of the VLPs. Proteins, nucleic acids, and PEG were each conjugated on the surface of VLPs through a Cu(I)-catalyzed reaction. Their system produced 0.3 mg/mL of VLPs that included up to 85% of the methionine analogs. However, the biological activity of GM-CSF was found to be reduced by 3–5 fold after being combined with VLPs. Patel and Swartz (2011) reasoned that the molecular crowding of scFv protein on the surface of VLPs and the curvature of the VLPs prevents GM-CSF to access GM-CSF receptors, leading to a decreased bioactivity of conjugated GM-CSF.

Altogether, CFPS systems have shown great potential to overcome the currently faced problems for *in vivo* virus and VLPs production. Further research and better optimization of CFPS protocols are needed to improve the robustness and potency of this technique when considering the creation of virus and VLPs. In the near future, CFPS systems may well replace the currently used cell-based methods at the production scale, given the advantages that the CFPS systems have over established methods.

## CFPS-Driven Biocontainment

Biocontainment is an aspect of biosafety concerning the organisms and species that can pose a risk to human health and ecology, and specifically covers their physical containment within secure areas, toward prohibiting their release into the wider community. Despite wide application and great successes, many current biocontainment strategies may not be effective enough for modern challenges, especially when concerning the release and spread of novel transgenes and/or transgenic organisms (Lee et al., 2018). Accordingly, there is a pressing need to develop new technologies to deal with biocontainment risks that threaten biosafety and biosecurity, and these technologies would ultimately serve to sharply decrease the potential severity and danger presented by genetically modified organisms. Among the many newer strategies that have been proposed to date, a number of them that involve cell-free systems have been suggested (Lee et al., 2018). The benefit of deriving proteins through CFPS systems in this way stems from their ability to remain abiotic, lacking most of the normal biotic processes of cells, while involving genes and DNA, which would be dividing, duplicating, and mutating. A number of utilities with these systems have already found success, including the biosynthesis of therapeutics and encapsulation of transcription/translation machinaries into vesicles. In all cases, the use of CFPS systems meant that the experimental process exhibited lower biosafety risk than with living systems, which may often be an undervalued aspect of cell-free systems in general.

Furthermore, the isolation of CFPS systems that are based on a xenobiological origin can restrict the spread of contamination that may result through the uptake/dispersal of transgenic materials and therefore compose another degree of security in their biocontainment level. CFPS systems have been employed to aid the incorporation of ncAAs via codon reassignment (Hong et al., 2014), which further increases biosafety through having modified organisms that have reassigned codon usage that are sufficiently alien to natural organisms so as to form a strong barrier against genetic contamination (Lee et al., 2018). This kind of xenobiological biosafety barrier is a deliberately sought out target for the field of xenobiology or xeno nucleic acids, and benefitting from use of cell-free systems underlines a clear and strong potential for these technologies to augment the biocontainment strategies of the future.

## Conclusion and Future Remarks

Synthetic biology is a modern and innovative scientific discipline with an aim to improve the existing industrial practices, addressing issues of poor yields and poor cost-to-product ratios, as well as the problems of current practices that inevitably damage our ecosystems through polluting acts. In any of these cases, it would be prudent to consider alternatives, and it is in synthetic biology that novel and alternative routes for the fabrication of many value-added products have been found with a compelling amount of accomplishments and an ever-fertile basis to grow future products and industries. In this context, we have reviewed and discussed CFPS, covering its numerous successes achieved to date and the wide-reaching potential for it to develop, as well as some of the necessary steps required.

CFPS systems offer a pronounced scientific impact that can drive development in many areas of high-throughput production by targeting the technology to aid the generation of valuable products that include proteins, therapeutics, and viruses/VLPs. For improving methods of protein production, CFPS systems have shown great efficiency to generate high levels of expression, purity, and yield, in addition to allowing the easier incorporation of labeled amino acids, factors that permit better NMR analysis of protein structures (Morita et al., 2003; Ozawa et al., 2004; Takai et al., 2008).

The relative ease of working with CFPS systems means that the time-consuming and laborious processes of cloning can be minimized, meaning that large-scale libraries of functional proteins can be made easier than before (Sawasaki et al., 2002; Liu et al., 2019; Rolf et al., 2019), aiding their functional study and use into biochips (He and Taussig, 2007; He et al., 2008), microarrays (Goshima et al., 2008), and continuous flow technologies (Shirokov et al., 2002). These findings indicate that CFPS systems could comfortably be expanded and used in the expression as well as testing of higher protein libraries designed for multi-well formats and experiments, allowing highly complex studies and high-throughput experiments that are assisted by this technology to work much faster that too at a lower cost, relative to current practices. Accordingly, an area of further research would be to test the full potential of CFPS systems and their applicability to assist other types of complex and high-throughput experiments.

Despite great progress in the biological domain of science, many proteins remain difficult to express *in vivo*, with issues in their complexity or toxicity as well as problems in solubilization/purification, especially for membrane proteins. We have discussed a number of difficult proteins that have already been produced with CFPS systems, including several restriction endonucleases (Goodsell, 2002), human microtubule binding protein (Betton, 2003), and cytolethal distending toxin (Ceelen et al., 2006), as well as some membrane proteins, including tetracycline pump TetA (Wuu and Swartz, 2008), vaccine antigens (Welsh et al., 2012; Lu et al., 2014), and the G-protein coupled receptor (Orbán et al., 2015). The expression of this range of difficult proteins is highly inspiring that it could be translated toward other challenging proteins. The importance that protein and enzyme products play in modern medicine and in the biological studies cannot be understated, and accordingly, it follows that one component of the progression for this technology may simply be to apply it rigorously to the proteins that remain too difficult to study. CFPS has the potential to drive new findings in various fields that could well revolutionize many medical treatments, as well as the biomedical studies of cancer, viral infections (via human receptors), and antibiotic resistance, among many others.

The currently used cellular lysates are derived from *E. coli*, wheat germ, rabbit reticulocytes, and insect cells (Kigawa et al., 2004; Liu et al., 2005; Schwarz et al., 2007), with manufactured systems of PURE also being used (Shimizu et al., 2001; Kuruma and Ueda, 2015). It would be of great interest to use other sources of cell-free materials and substrates, especially toward addressing one of the problems in CFPS systems that concerns the post-translational modifications of products. These modifications include glycosylation, disulfide bonding, and correct protein folding. Further developments in CFPS systems would accordingly involve the testing and assay of new cellular lysates, toward identifying the best ones for all of the desired modifications possible, a project that could eventually develop into an *in silico* form as with numerous other protein synthesis and design assisting platforms.

Currently, cell-free systems are being used for cost-effective detection of Ebola, Zika, and dengue viral strains (Pardee et al., 2014, 2016; Gootenberg et al., 2017; Khambhati et al., 2019b), diseases that often affect poorer areas of the world with challenging accessibility issues. The invention of cheaper and portable tests that employ cell-free systems can revolutionize the manner in which these lethal diseases can be detected and dealt with, reducing their burden on human health. We believe that, in the future, CFPS will be able to address these diseases more sternly, as the damage that they do to people and communities is too high. Having realized these powerful methods for detecting these diseases, we appreciate the potential for how these can be adapted against the new viral outbreaks in future, to assist in better disease management than before. This should also be expanded for the other diseases of the world that remain difficult to diagnose and treat, where ever appropriate.

In other areas, CFPS systems have been used in a wide range of experiments, including the production of proteins that incorporate toxic amino acids such as canavanine (Worst et al., 2015), integration of orthogonal genetic codes (Chemla et al., 2015; Des Soye et al., 2015), production of therapeutic medicines (Zawada et al., 2011), and the assembly of bacteriophages (Shin et al., 2012), among many others. Many of these experiments give clear indications on the future work that must be performed for CFPS systems. In one avenue, cell-free TX–TL systems have allowed the incorporation of L-canavanine and L-hydroxy-lysine into proteins, opening the door for the future examination of these and other amino acid replacements. The benefit here is that, with the expansion of basic language of proteins, they are now capable of possessing novel functionalities that are otherwise toxic to living cells, opening a whole new world for modified protein and enzyme functionalities that haven't been considered before. The cell-free generation of cIFN-α in good yields is another exciting result, one that also demands future development to confirm the potential for it to be a novel anti-cancer treatment beyond its current proficiency *in vitro* (El-Baky et al., 2011).

There is an exigency for more and better drugs against the myriad forms of cancer, and this result demonstrates that CFPS systems could be well-adapted for their synthesis or for improving the existing methods. A related result that should be explored further is the cell-free synthesis of viruses/VLPs. Viruses and VLPs can be used to develop experimental gene therapy treatments, drug delivery, diagnostic tools, and nanotechnology applications (Shirbaghaee and Bolhassani, 2016). CFPS systems have demonstrated extraordinary competence for generating viruses/VLPs, exceeding some *in vivo* techniques, and the importance and potential of these species is such that it is likely that cell-free systems could replace cell-based methods, with a future aim of the field to extend this progress to industrial production scales (Bundy et al., 2008; Patel and Swartz, 2011).

Within all of these aspects, the use of CFPS systems has enabled biologists to advance in each of these distinct areas, discovering new results and findings. In general, we believe that this range of studies that have been benefited by cell-free expression systems offer a very promising belief that these systems can be redeployed into many other scientific studies, offering advantages that permit countless other interesting and compelling experiments to be performed, more quickly and at a lower cost, similarly to the ones that we discussed. Even in the smallest of cases, if the use of these systems can save money and time, it may well open the door for lowering the barriers to allow entry of many scientists and their projects, offering greater diversity of ideas and experiments to be possible. Lastly, we believe that the CFPS systems that we have discussed have already realized numerous successes and with the current rate that modern science and synthetic biology is growing, it is clear that novel developments and innovations must follow. We must extrapolate these successes to address many existing world issues in novel, safer, more efficient, and greener ways, to benefit the health of the planet, and ultimately remove our reliance on non-renewable and polluting sources of valuable products and energy.

## Author Contributions

KK, GB, NG, DB, and VS have designed and written the manuscript. VK and DB have proofread and given comments as well as suggestions. VS has supervised and finalized the manuscript.

### Conflict of Interest

The authors declare that the research was conducted in the absence of any commercial or financial relationships that could be construed as a potential conflict of interest.

## Abbreviations

ATP: Adenosine triphosphate
CFPS: Cell-free protein synthesis
CHO: Chinese hamster ovary
GM-CSF: Granulocyte macrophage colony-stimulating factor
IRES: Internal ribosome entry sites
LETs: Linear expression template
MAGE: Multiplex automated genome engineering
ncAAs: Non-canonical amino acids
NMR: Nuclear magnetic resonance
nsAAs: Non-standard amino acids
pAcF: p-acetyl-L-phenylalanine
PEG: Poly-ethylene glycol
PEP: Phosphoenolpyruvate
PISA: Protein *in situ* array
pPaF: p-propargyloxy-L-phenylalanine
PSBS: Photosystem II subunit S
PURE: Purified recombinant elements
RF1: Release factor 1
scFv: Single-chain antibody variable fragment
sfGFP: Super folder green fluorescent protein
TX–TL: Transcription–translation
UAAs: Unnatural amino acids
USD: United States dollar
VLPs: Virus-like particles
WGE: Wheat germ extract
YCE: Yeast cell extracts
IGF-I: Insulin-like growth factor-I.
